# Bridging Space Perception, Emotions, and Artificial Intelligence in Neuroarchitecture

**DOI:** 10.3390/brainsci16020131

**Published:** 2026-01-26

**Authors:** Avishag Shemesh, Gerry Leisman, Yasha Jacob Grobman

**Affiliations:** 1 Movement and Cognition Laboratory, Department of Physical Therapy, University of Haifa, Haifa 3103301, Israel; avishag.shemesh@gmail.com; 2 National Institute for Brain and Rehabilitation Sciences, Gilbert, AZ 85013, USA; 3 Clinical Neurophysiology, Institute for Neurology and Neurosurgery, University of the Medical Sciences of Havana, Havana 11300, Cuba; 4 Faculty of Architecture and Town Planning, Technion–Israel Institute of Technology, Haifa 3200003, Israel; yasha@technion.ac.il

**Keywords:** neuroarchitecture, evidence-based design, environmental neuroscience, built environment, cognition, space geometry

## Abstract

In the last decade, the interdisciplinary field of neuroarchitecture has grown significantly, revealing measurable links between architectural features and human neural processing. This review synthesizes current research at the nexus of neuroscience and architecture, with a focus on how emerging virtual reality (VR) and artificial intelligence (AI) technologies are being utilized to understand and enhance human spatial experience. We systematically reviewed literature from 2015 to 2025, identifying key empirical studies and categorizing advances into three themes: core components of neuroarchitectural research; the use of physiological sensors (e.g., EEG, heart rate variability) and virtual reality to gather data on occupant responses; and the integration of neuroscience with AI-driven analysis. Findings indicate that built environment elements (e.g., geometry, curvature, lighting) influence brain activity in regions governing emotion, stress, and cognition. VR-based experiments combined with neuroimaging and physiological measures enable ecologically valid, fine-grained analysis of these effects, while AI techniques facilitate real-time emotion recognition and large-scale pattern discovery, bridging design features with occupant emotional responses. However, the current evidence base remains nascent, limited by small, homogeneous samples and fragmented data. We propose a four-domain framework (somatic, psychological, emotional, cognitive-“SPEC”) to guide future research. By consolidating methodological advances in VR experimentation, physiological sensing, and AI-based analytics, this review provides an integrative roadmap for replicable and scalable neuroarchitectural studies. Intensified interdisciplinary efforts leveraging AI and VR are needed to build robust, diverse datasets and develop neuro-informed design tools. Such progress will pave the way for evidence-based design practices that promote human well-being and cognitive health in built environments.

## 1. Introduction

Neuroarchitecture represents an interdisciplinary domain that integrates principles from neuroscience and architecture to investigate how individuals perceive and respond to various spatial environments. Insights into the cognitive and emotional processing of space have profound implications for the design of environments tailored to specific functions and user needs. Traditional architectural paradigms often overlook these psychological and physiological considerations, focusing instead on technological advancements or adherence to regulatory standards. The objective of this review is to synthesize research at the intersection of architecture and neuroscience, offering a cohesive framework for interpreting current findings and identifying persisting gaps in knowledge. In the context of rapid advancements in artificial intelligence, the scarcity of empirical data in this area becomes increasingly evident, highlighting the necessity for systematic knowledge generation. As AI enables more widespread and autonomous design practices, there is a growing imperative to produce robust empirical evidence to inform and optimize design decisions. Prior narrative reviews have largely summarized isolated findings without mapping specific architectural variables to measurable physiological and behavioral outcomes or detailing how VR and AI can operationalize such mappings. To address this gap, the present review explicitly integrates immersive VR protocols, multimodal physiological sensing, and AI-based analytics within a single methodological and theoretical framework. This contribution aims to provide reproducible guidance for future neuroarchitectural studies and clarify how design parameters can be linked to the SPEC domains (somatic, psychological, emotional, cognitive).

The field of neuroarchitecture has grown significantly in the last decade, with researchers establishing important connections between architectural features and neural processing [1,2,3]. Contemporary research increasingly emphasizes the necessity for empirically valid experimental designs and a broader recognition of embodied experiences that extend beyond purely visual aesthetic judgments [4,5]. Contrary to the perception of a dichotomy between embodiment and aesthetic-focused research, emerging evidence suggests that aesthetic responses can be physiologically manifested [6]. The rapid advancement of Virtual Reality (VR) and artificial intelligence (AI) highlights a significant gap in empirical data regarding these aspects, emphasizing the critical need for systematic knowledge accumulation to inform evidence-based design practices. As AI technologies democratize the design process and encourage a “do-it-yourself” ethos, the imperative to generate robust empirical knowledge becomes even more pronounced, ensuring that accessible and scientifically grounded guidance is available for effective architectural design.

One of the main foci of neuroarchitectural research is the exploration of how architectural space affects four fundamental domains of human experience: somatic (physiological), behavioral/psychological, emotional, and cognitive, collectively referred to as the “SPEC”. It attempts to tie specific spatial features (e.g., ceiling height, natural light, curvature) to activity in the anterior cingulate cortex, parahippocampal place area, and other circuits that govern emotion, stress, and attention [7,8,9,10]. VR seems to accelerate this work: hybrid VR electroencephalography (EEG) platforms can collect and analyze high-frequency brain, physiological, and behavioral data in immersive, controllable spaces at unprecedented scale and speed [7]. Despite the accumulation of knowledge and the emergence of new technologies, the empirical evidence base remains insufficient. AI systems operating in this domain are only as effective as the data repositories from which they draw. In domains where empirical evidence is sparse or biased, algorithms inherit those blind spots, as starkly illustrated by documented inequities in medical AI systems [8]. Learning-space design is a case in point: while multi-level studies show that classroom geometry, daylight, acoustics, and color together explain up to 16% of the variation in pupils’ learning rates [9], the geographic and demographic coverage of such datasets remains patchy. We do not possess sufficient empirical data regarding: (a) the understanding of key criteria that influence our limbic system and emotional response in space, and (b) the influence of space as a complex on our limbic system and our emotions.

In addition, previous reviews have primarily cataloged correlations between design parameters and neural responses without integrating how VR and AI methods can systematically capture multisensory, embodied, and affective aspects of space perception. To close this gap, the current review positions VR-based experimentation and AI-driven analytics as complementary tools for establishing reproducible, quantitative links between architectural variables and the SPEC domains, thereby extending neuroarchitectural inquiry beyond descriptive observation toward predictive modeling.

This article provides a comprehensive review of research at the intersection of neuroscience and architecture, with a particular emphasis on the application of AI and VR technologies. The topics addressed include the integration of neuroscience and architectural principles, the utilization of AI to assess emotional responses within VR environments, and the investigation of the emotional impact elicited by architectural spaces and broader environmental contexts. Furthermore, the review examines studies exploring the autonomic nervous system’s role in emotional experience, particularly through the use of VR, as well as research on visual perception and its affective dimensions within architectural settings. The use of AI in the past several years has also produced new research, contributing data to this knowledge scope, as we later present. Based on this synthesis, we contend that addressing current gaps in the literature requires intensified research efforts focused on leveraging AI’s capacity to collect, analyze, and synthesize empirical data. Such efforts are essential for advancing our understanding of spatial perception, accurately measuring and recognizing emotional responses, and establishing comprehensive criteria for architectural environments that have been insufficiently explored to date.

## 2. Literature Search Methodology and Selection Criteria

The primary databases utilized for this review included Web of Science, Scopus and PubMed. Supplementary searches were conducted using APA PsycINFO, IEEE Xplore, ACM Digital Library, Crossref, and Google Scholar. The initial search strategy employed the term “neuroarchitecture.” Additional screening involved the use of various terms in different combinations, including “neuroscience” and “architecture”; “emotional impact” and “buildings” or “architectural spaces”; and “architectural space perception” and “emotional response.” The search was further refined by incorporating additional keywords (to enlarge the scope), such as “autonomic nervous system”, “physiological response”, “VR”, and “AI.” The objective was to identify relevant literature outside the immediate field of neuroarchitecture and to recognize empirical studies, both within and beyond the architectural domain, with a particular focus on the gap between technological advancements and their implementation in neuroarchitectural research.

**Figure 1:**
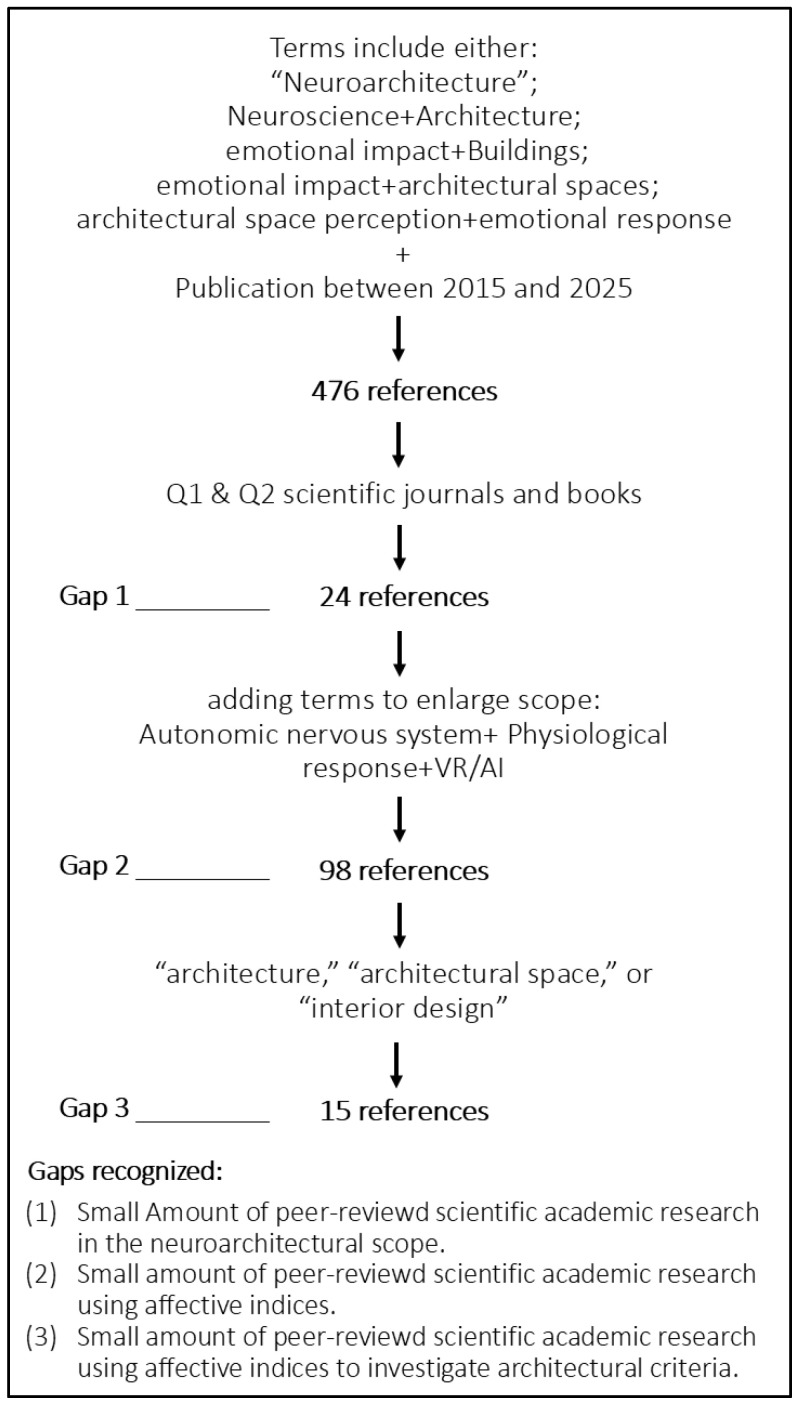
Literature search methodology and selection criteria.

Inclusion criteria were as follows: (1) publication in peer-reviewed Q1 or Q2 scientific journals and books (no conference proceedings) (2) Publication between 2015 and 2025, and (3) studies employing the search terms in various combinations. The initial search yielded 476 references, which were subsequently narrowed down based on these criteria to 24 references. Adding the mentioned keywords enlarged the database to 98 (From them, only 15 references mention “architecture,” “architectural space,” or “interior design” in their title/abstract). Data extracted from each study highlighted key findings and their implications for the respective topics. Thematic analysis revealed three primary categories: (1) core components of neuroarchitecture, (2) the use of physiological sensors and the generation of empirical data, and (3) the integration of neuroscience and AI to advance the field of neuroarchitecture. The discussion synthesizes these themes, culminating in a unified conclusion. Figure 1 summarizes the literature search methodology and selection criteria, highlighting gaps along the process.

## 3. Background and Core Concepts

### 3.1. Neuroscience and Architecture

#### 3.1.1. Neuroscience and Architecture: Background

In recent years, the interdisciplinary connection between architecture and neuroscience has grown, supported by technological advances that let researchers probe how the mind processes environmental stimuli. Neuroscientific tools such as electroencephalography (EEG) and functional magnetic resonance imaging (fMRI) reveal brain responses to architectural images and spaces, while VR platforms provide realistic, multisensory scenes that deliver ecologically valid tests of spatial cognition [10].

A persistent methodological hurdle is that most neuroimaging devices require participants to remain still; natural movement through 3D spaces introduces motion artifacts that can mask perceptual or cognitive signal [11]. Even so, less expensive VR kits, accessible 3D modeling software, and closer cross-disciplinary collaboration have driven a surge in studies on the emotional and cognitive impact of built environments [12,13].

Within this emerging field of neuroarchitecture, researchers have examined the affective power of places at both room- and city-scales [14,15] and highlighted the public-good potential of such work [16]. At the same time, authors argue for more rigorous, experimentally grounded methods that move beyond traditional “brain-in-a-scanner” set-ups so the field can mature into a full experimental science [12,13]. Notably, one gap remains: systematic tests of how specific geometric features in 3D environments shape emotion and cognition.

#### 3.1.2. Neuroscience and Architecture: Recent Research

Current scholarship in neuroarchitecture coalesces around two interrelated agendas: (a) isolating the environmental attributes that drive users’ perceptual, cognitive, and affective responses, and (b) deploying neurophysiological methods to evaluate how those attributes modulate experience in real or in simulated settings. Early work integrating principal-component analysis with fMRI established three higher-order experiential dimensions: coherence, fascination, and hominess, which map onto dissociable activity patterns in the visual cortex [17]. Methodological critiques soon followed, cautioning that an overemphasis on aesthetic appraisal risks eclipsing the embodied, action-oriented nature of architectural experience [5]. Systematic reviews confirm that the field still draws on a heterogeneous theoretical repertoire, signaling both conceptual richness and the need for stronger synthesis [3].

Subsequent studies have begun to interrogate inter-individual variability. For example, voxel-based morphometry revealed that regional gray-matter volume modulates the extent to which the personality trait of openness shapes judgments of beauty and pleasantness in interior spaces [18]. Parallel EEG inquiries show that beta-band power predicts spatial satisfaction, whereas alpha-band dynamics correlate with aesthetic preference [19]. In immersive virtual-reality wayfinding tasks, higher beta-band activity over the right temporal cortex correlates with better wayfinding efficiency [20], while frontal-alpha asymmetry differentiates preferred lighting conditions [19] and occipital-gamma power increases in biophilic hospital rooms [21].

Spatial cognition has likewise been linked to structural brain indices: larger parahippocampal volume is associated with superior memory for complex layouts [22]. Cultural context also matters; default-mode-network (DMN) activation diverges when observers view sacred versus secular spaces [23]. Multimodal approaches that combine EEG with eye tracking [24], mobile brain/body imaging, and wearable caps [25] now capture neural responses during naturalistic navigation. Complementary physiological probes have extended the design space to curvature [26,27,28,29], scale [26], color-scheme evaluation [30] and room acoustics [31].

A second strand of research examines how neurophysiological metrics predict users’ experience of function-specific settings. Resting-state fMRI combined with graph theory shows hippocampal connectivity tracks the intelligibility of urban street networks [32]. In another research, a space for work, subdued colors were preferred, while for leisure, gray and bright colors were preferred [33,34]. Shemesh et al. have divided different geometries of spaces according to suggested uses in addition to questionnaires [35]. Nevertheless, the examination of emotional reaction to different uses of spaces remains rare.

Collectively, these findings underscore the promise of neuroscientific tools for elucidating the mechanisms through which the built environment shapes perception, emotion, and behavior, while highlighting the imperative for more integrative frameworks that bridge aesthetics, embodiment, and situated action.

In recent VR-based experiments, environmental design manipulations (such as changes in spatial geometry, lighting, or biophilic elements) have been systematically paired with standardized behavioral and physiological measures covering all SPEC domains [36]. For example, altering room geometry or illumination in an immersive setting can influence approach-avoidance behaviors and navigation (wayfinding) efficiency, while concurrently modulating bodily and neural responses like heart-rate variability and EEG band power [37]. In one virtual hospital wayfinding study, a more visually enriched corridor design (enhanced lighting, color, and signage) led participants to orient more effectively and also elicited stronger occipital EEG engagement during decision points [26]. Likewise, highly stimulating VR scenarios have been linked to synchronized somatic and neural responses- for instance, a rollercoaster VR experience induced lower high-frequency HRV alongside suppressed alpha-band EEG power under high emotional arousal [37].

By reporting such behavioral outcomes, physiological indices, and neural metrics in tandem, researchers can directly map specific environmental features to somatic stress indicators, emotional states, cognitive engagement, and overt behavior, making results more comparable across studies. Architects, neuroscientists, and data scientists seeking reproducible frameworks (SPEC) may benefit this accumulated knowledge. To facilitate rigor and reproducibility in VR-based neuroarchitectural studies, please explicitly report core VR parameters and data-quality controls: display type and stereoscopy, field of view, frame rate/latency, locomotion interface, audio rendering, calibration of scale/lighting, and motion, artifact handling/synchronization with physiological signals. Stating these elements allows results to be compared across labs and clarifies which spatial manipulations (e.g., curvature, height, enclosure) responsible for observed changes in behavior and physiology. In Section 3.2.3, the visual perception of virtual spaces in neuroarchitecture research will be discussed in further detail.

#### 3.1.3. Neuroscience and Architecture: Summary

The field of neuroarchitecture has grown significantly since 2020. Out of a total of 476 articles, 287 are from the year 2020 and after, with researchers establishing important connections between architectural features and neural processing. These publications demonstrate the value of interdisciplinary approaches combining neuroscientific methods with architectural analysis. Within this framework, contemporary scholarship can be organized into two complementary strands: (i) isolating the environmental components that modulate user responses and (ii) evaluating how whole-space configurations support specific activities through advanced neuroimaging and psychophysiological tools. Results of the first strand underscore the multiplicity of design variables that enter the neural calculus of architectural appraisal. The second research strand examines how holistic environments support particular functions. Together, these two strands demonstrate a shift from cataloging aesthetic impressions to formulating informed design principles that encompass action, affect, and multisensory integration.

This recent growth reflects both expanded methodological capacity and an urgent need for transparent reporting and common metrics. Establishing minimal reporting standards (VR configuration, physiological acquisition settings, preprocessing, and synchronization) will strengthen reproducibility and help the field progress from descriptive associations toward predictive, design-relevant models.

### 3.2. Visual Perception of Spaces and Neuroarchitecture

#### 3.2.1. Visual Perception of Spaces: Background

The investigation of visual perception mechanisms in architectural spaces represents a fundamental cornerstone for advancing neuroarchitecture research methodologies and expanding empirical knowledge bases [27]. Recent developments in this interdisciplinary field demonstrate that by understanding how the human brain processes architectural visual stimuli, researchers can develop more precise experimental protocols, enhance data collection techniques, and establish comprehensive databases that bridge neuroscience and architectural design. The convergence of neuroimaging technologies, virtual reality systems, and behavioral measurement tools has enabled insights into how specific brain regions respond to architectural features, suggesting that visual perception operates through hierarchical processing stages where early-detected spatial features significantly influence overall environmental interpretation. This synthesis of visual perception research not only illuminates the neural mechanisms underlying spatial experience but may also provide architects and designers with evidence-based frameworks for creating environments that optimize human cognitive and emotional responses. The conceptual foundation of neuroarchitecture research begins with understanding how visual perception of space occurs. This framework draws on established neuroscientific principles that describe visual processing as a hierarchical system of interconnected stages. As Shimojo, Paradiso, and Fujita explain, visual perception unfolds through a series of computational levels: lightness computations occur at intermediate stages, following early contour extraction but preceding surface color analysis [28]. This progression indicates that the features first registered in a spatial scene exert a disproportionate influence on how the brain subsequently interprets the overall environmental experience.

Contemporary neuroarchitecture research has expanded this understanding by identifying specific brain regions that respond to architectural visual stimuli [29]. The field has demonstrated that perception of built environments activates distinct neural networks, including the parahippocampal place area (PPA), occipital place area (OPA), and retrosplenial complex (RSC), which collectively process visual complexity [38], spatial boundaries [39,40], and environmental landmarks [41]. These discoveries reveal that both real and virtual architectural experiences can trigger consistent neural responses, providing validation for laboratory-based research methodologies. The integration of embodied cognition theories further enriches our understanding of architectural visual perception [5,33]. The enactive approach to architectural experience emphasizes the profound connectedness between organism and environment in active, dynamic relationships, challenging traditional models that treat perception as passive information processing. This perspective has proven particularly valuable for developing systematic accounts of architectural experience that can guide scientific experimentation and provide consistent frameworks for interpreting research results. The embodied framework naturally aligns with the SPEC domains, somatic (sensorimotor), psychological (behavioral intent), emotional (affective resonance), and cognitive (spatial reasoning), providing an operational structure for interpreting embodied perception data.

Research into the neural mechanisms of architectural form perception has revealed processing systems that respond to geometric and spatial properties of built environments [34]. Investigations by Banaei and colleagues using virtual reality and EEG have demonstrated that the anterior cingulate cortex (ACC) shows significant activation when individuals navigate environments with curved architectural features [12,42]. This finding establishes the ACC’s central role in processing architectural form and spatial experience, providing concrete evidence for how geometric properties influence neural activity patterns. Curvature-related ACC activation has also been correlated with self-reported pleasantness and reduced sympathetic-arousal indices, linking geometric parameters to both neural and autonomic components of the SPEC framework.

The importance of understanding these neural mechanisms extends beyond basic research to practical applications in architectural design. Studies examining sensorimotor brain dynamics have shown the way our brain processes movement changes depending on how the design of a space allows or restricts our actions [43]. These findings suggest that cognition is inherently related to potential body movement, positioning action and perception as interrelated processes that actively influence environmental experience. For architects, this research indicates that spatial design should consider the continuity of movement and the unfolding prediction of affordance worlds that users construct as they navigate spaces. Integrating these insights with quantitative movement-tracking or motion-capture data can enrich neural analyses, allowing future studies to examine how predicted affordances correspond to measurable motor outcomes and user comfort.

Mirror neurons have emerged as particularly significant components in architectural perception research [29]. These specialized neural systems generate empathetic responses to environmental surroundings, contributing to the emotional and cognitive experiences that architectural elements like lighting, color, and spatial layout can evoke. The discovery of mirror neuron involvement in architectural perception has opened new avenues for understanding how built environments can influence human behavior and well-being through neurobiological mechanisms. In neuroarchitectural contexts, mirror-neuron activation may reflect embodied simulation processes through which occupants ‘feel into’ spatial form; documenting this relationship via synchronized EEG-EMG recordings could quantify embodied resonance objectively.

Eye-tracking studies have provided particularly valuable insights into visual exploration patterns in architectural spaces [44,45]. Research by Wang, Zhang, and Zhou using VR and eye-tracking found that natural elements and open spaces possess restorative qualities, unlike architectural elements such as architectural corridors and building façades [46]. Another research by Li et al. [47] found that forests that you can easily see through, because of tree type and lighter understory, look safer and more appealing to visitors. These findings provide concrete guidance for designing visually comfortable environments and demonstrate how technological advances enable precise measurement of visual attention patterns in spatial contexts. Still, the way our eye scans different spaces and which elements in the architectural designed environment receive “priority” over others has yet to be explained. Future investigations can address this open question by integrating gaze-tracking data with neural and autonomic measures, such as concurrent EEG-pupilometry, to model the temporal coupling between attentional shifts and affective appraisal in immersive spaces.

#### 3.2.2. Visual Perception of Spaces with VR

Virtual environments (VEs) offer researchers the ability to design dynamic, immersive settings that allow for precise manipulation of variables while holding other design features constant, thereby reducing experimental noise [48]. This methodological advantage is particularly valuable when real-world environments present excessive or uncontrollable information. Through VEs, researchers can create specific perceptual conditions for users and analyze discrete components of human skills- such as spatial cognition and task performance [48]. Recent empirical studies have compared participant performance, perception, and sense of presence in physical versus immersive virtual environments, validating the use of VEs for controlled experimentation [48]. Educational institutions have increasingly adopted VR to explore the relationship between space and society, develop social topographies, and assess the impact of VR-based learning activities [49]. These initiatives include VR health-education games, medical-training scenarios, and comparative studies on learning outcomes across various disciplines.

Controlled VR studies that manipulate depth cues, stereoscopy, or illumination have demonstrated that even subtle differences in visual rendering parameters can alter physiological markers such as heart-rate variability and frontal-alpha power, underscoring that accurate reporting of these technical parameters is essential for reproducibility and interpretation. Controlled comparisons consistently show strong correspondence between behavioral metrics (navigation time, error rate) and neural indicators (frontal-alpha asymmetry, parietal beta power) obtained in virtual and real spaces, strengthening confidence in the ecological validity of VR methods [50].

In healthcare, VR combined with physiological sensors enables the investigation of neural mechanisms underlying perception and action, with applications in enhancing mobility and reducing fall risk [51]. VR interventions have also proven useful for anxiety reduction [52] and for promoting physical activity through exergaming, although the effects on children and adolescents remain under careful evaluation [53]. These findings illustrate the potential of immersive systems for examining somatic and emotional components of the SPEC framework, for example, modulation of autonomic markers (heart-rate variability, EDA) alongside neural activation patterns in therapeutic or rehabilitative contexts.

#### 3.2.3. Visual Perception of Virtual Spaces in Neuroarchitecture Research

Virtual reality technologies have revolutionized neuroarchitecture research by enabling controlled manipulation of architectural variables while maintaining immersive spatial experiences [29]. Studies examining how VR environments activate the same brain regions as real architectural spaces have validated the use of virtual environments for research purposes, significantly expanding the range of experimental conditions that researchers can investigate safely and cost-effectively. Across such studies, overlap between parahippocampal and retrosplenial activation in physical versus virtual settings demonstrates that spatial-layout processing is preserved across modalities, providing a strong empirical foundation for extending findings from virtual laboratories to real-world design.

The integration of multiple measurement techniques has become increasingly sophisticated, with researchers combining neuroimaging, behavioral measures, and environmental sensors to create comprehensive datasets. Researchers have documented how methods such as VR, eye-tracking, and neuroimaging can be combined to examine how spatial layout, color, and lighting [54] shape perception and affect in architectural spaces [55]. Mostafavi argues that combining immersive VR with neuro measures (e.g., EEG, eye-tracking, GSR) offers a robust “triangulation” framework to evaluate architecture by capturing users’ cognitive, emotional, and physiological responses [56]. Such multimodal synchronization, linking gaze position, neural oscillations, and galvanic responses, directly operationalizes the SPEC domains and supports development of predictive models of user experience. Parallel activation patterns across real and virtual settings suggest that well-calibrated immersive environments can faithfully reproduce core perceptual dynamics, validating VR as an ecologically valid proxy for built-environment studies. To maintain consistency with reproducible-design principles, future reports should describe the visual stimuli in quantifiable terms (field of view, luminance, color-temperature range, and stereoscopic rendering) so that visual-processing results can be replicated and compared across laboratories.

The application of visual perception research spans diverse architectural contexts, each contributing unique insights to the broader understanding of human–environment interactions. Educational environments have received considerable attention, with studies by Barret et al. demonstrating how daylighting conditions influence visual perception and comfort in classrooms [9]. These investigations have shown that appropriate daylight levels improve visual clarity and comfort, directly supporting student well-being and academic performance through measurable neurophysiological mechanisms. Future classroom VR simulations could standardize illuminance, color temperature, and contrast parameters, allowing controlled tests of how lighting geometry modulates attentional and emotional engagement, an essential step toward design reproducibility.

Residential architecture research has focused particularly on the psychological impacts of visual access to natural elements. An examination of window views in high-rise apartments has revealed that views of natural elements enhance visual comfort and restorative experiences, highlighting the importance of visual connections to nature in residential design [57]. These findings demonstrate how specific visual features can be quantified and optimized to promote psychological well-being through evidence-based design approaches. Integrating VR scenarios of differing window orientations and vegetation density with physiological measures could systematically quantify restorative effects and inform evidence-based biophilic guidelines.

Urban environments present complex challenges for visual perception research, as documented in studies of streetscape perception by Gjerde and Vale. Their empirical investigation of building height and façade articulation effects on visual perception has demonstrated that moderate building heights and articulated façades are generally perceived as more visually appealing and comfortable [58]. To strengthen cross-study comparability, future VR streetscape experiments should report spatial-scale calibration, viewpoint motion constraints, and luminance distribution, which strongly influence façade-preference outcomes.

#### 3.2.4. Visual Perception in Neuroarchitecture Research: Summary

A multisensory perspective has challenged traditional approaches that focused primarily on visual perception in isolation [33]. The enactive approach to architectural experience emphasizes embodiment, motivation, and affordances as interconnected components of spatial experience. The integration of ecological psychology principles has provided additional theoretical grounding for understanding multisensory architectural experience [5]. This approach addresses limitations of traditional experimental methods by emphasizing naturalistic interaction with architectural surroundings and recognizing the active role of the perceiver in constructing environmental meaning. Recent research has also examined how different sensory modalities contribute to spatial navigation and wayfinding [29]. Studies have shown that architectural elements facilitating orientation and navigation activate specific neural networks related to spatial cognition, with implications for designing environments that support efficient and comfortable movement patterns.

In summary, VR research that integrates multisensory cues, visual, auditory, and haptic, within quantified experimental parameters can directly test predictions across all SPEC domains, thereby linking perceptual fidelity to emotional and cognitive outcomes.

### 3.3. Visual Perception and Emotional Affect Research Using AI

The advancement of visual perception research in neuroarchitecture continues to generate new methodological possibilities and research directions [42]. Comprehensive scoping reviews of the field have identified both limitations and benefits of current approaches, highlighting the need for continued development of research methods that can capture the full complexity of architectural experience while maintaining scientific rigor. Emerging technologies in neuromorphic engineering and biologically plausible computing systems offer new possibilities for understanding and modeling architectural perception [59]. These developments may enable more sophisticated simulation of neural processes underlying spatial experience, potentially leading to computational models that can predict human responses to architectural designs before construction. The integration of artificial intelligence and machine learning approaches with traditional neuroscience methods presents additional opportunities for analyzing complex datasets generated by multisensory research [44]. Advanced data analysis techniques may reveal patterns in neural responses to architectural features that were previously undetectable, expanding our understanding of the mechanisms underlying spatial perception and preference. Future AI-based analyses should specify model type (e.g., CNN, LSTM), training-data composition, validation procedure, and feature-extraction pipeline so that predictive results are reproducible and comparable across laboratories. AI and VR are contributing to the neurosciences by letting researchers evoke, measure, and model brain and body reactions in controllable immersive worlds. In VR, every visual angle, light level, or spatial proportion can be manipulated, while AI algorithms may harvest the resulting torrents of physiological and behavioral data in real time. The papers below capture three complementary strengths of the VR-AI combination: (1) precise emotion decoding, (2) fine-grained autonomic nervous system (ANS) tracking, and (3) rapid design-feedback loops that turn neuroscientific insight into architectural guidance. Linking these functions to the SPEC framework clarifies their scope: emotion decoding reflects the emotional domain, ANS tracking the somatic, and design feedback the cognitive and psychological components that together close the loop between perception and design.

Headsets now ship with inward-facing cameras and inertial sensors, giving computer-vision models a direct view of a user’s micro-expressions and movements [45]. A MobileNet-V2 network running inside Unity recognized neutral, happy, sad, and surprised faces with high accuracy in a small cohort, even though anger and fear remained harder to separate—demonstrating that reliable facial analytics are feasible in everyday VR gear [60]. Deep-learning tools are getting better at solving a common problem for architects: EmojiHeroVR for instance is using several camera views and image processing to read expressions even when half the face is covered. This makes it possible to give users personalized emotion feedback during virtual design reviews [60]. To ensure reproducibility, reports of such emotion-recognition systems should include camera resolution, frame rate, illumination calibration, and facial-landmark-tracking error so that accuracy metrics can be independently verified.

Emotion recognition is not limited to images. A systematic review covering 13 studies shows that AI classifiers can reach real-time accuracy with EEG streams inside multisensory VR scenarios, opening the door to group-level affect monitoring during collaborative walkthroughs [61]. Multimodal approaches boost robustness: a multi-scale attention LSTM that fuses skin conductance, temperature, and motion signals reliably predicts valence and arousal across varied scenes [62] and a biosignal-fusion network combining heart rate, electrodermal activity, and respiration reaches similarly high precision [63]. Such architectures may give architects a continuous, objective read-out of how specific volumes, colors, or geometries feel to occupants. These multimodal pipelines operationalize SPEC variables by mapping somatic (physiological), emotional (valence/arousal), and cognitive (interpretive) responses within unified datasets, thereby enabling data-driven architectural evaluation.

Because a user in VR can physically turn, lean, or even walk while the surrounding world stays perfectly aligned, researchers can probe the ANS under naturalistic yet repeatable conditions. Immersive seaside, forest, or city simulations raise sympathetic drive in patients with disorders of consciousness, as indexed by electrodermal activity spikes [64]. At the opposite end of the health spectrum, a randomized trial showed that ten minutes of VR-based meditation improved heart rate variability indices, reduced self-reported stress, and enhanced sleep quality in nursing students [65]. A broad systematic review of built-environment VR exposures confirms the pattern: virtual settings reliably shift HRV, galvanic-skin responses, and blood pressure [66], validating VR as a stress-science laboratory. For architectural research, these findings imply that controlled manipulations of spatial geometry or lighting can serve as standardized stressors or relaxers, enabling quantifiable testing of restorative-design hypotheses.

AI multiplies the value of these physiological streams. A survey of peripheral-nervous-system applications charts how deep neural networks segment nerve imagery, predict neuropathic pain, and even estimate autonomic balance from raw electrocardiograms [67]. High-resolution capacitive ECG combined with facial and respiration tracking can map millisecond-level links between a fleeting emotion and its cardiovascular echo, an approach that would be unwieldy without machine-learning-based signal cleaning and feature extraction [68]. Experimental manipulations of VR object size reveal why such fidelity matters: simply enlarging virtual furniture heightens anxiety and heart rate, proving how small geometric tweaks translate into visceral change [69]. Meta-analytic work shows that VR stressors also trigger reliable adrenocortical responses, reinforcing their ecological validity [70]. Crucially, AI-augmented VR interventions can reverse these effects, lowering ANS activation when psychological support is blended into the simulation [71]. Integrating these AI-driven analytics with standardized reporting of hardware, sampling rate, and algorithm parameters will strengthen cross-study comparability and support meta-analytic synthesis within neuroarchitectural research.

Artificial intelligence now makes it possible to analyze large datasets- such as physiological signals- that until recently demanded labor-intensive statistical work and hand-crafted algorithms. This capability opens multidisciplinary research to broader, more accessible knowledge. Therefore, we can expect faster development of the neuroarchitecture field and a narrowing of existing knowledge gaps.

In conclusion, transparent documentation of AI model specifications and VR acquisition settings will allow future studies to build cumulative, reproducible databases, advancing the transition of neuroarchitecture from descriptive to predictive science grounded in the SPEC framework.

## 4. Current Evidence and Thematic Synthesis

Of the 476 articles retrieved, 15 qualified as empirical investigations in neuroarchitecture, while a further 83 were classified as studies that offer promising avenues for follow-on work likely to narrow existing knowledge gaps- gaps still largely defined by the initial 15 papers. Although this distribution is unsurprising given the topic-focused search strategy, the current evidence base remains too limited to construct a robust dataset capable of underpinning emotion-oriented design. These findings therefore underscore the need to broaden the search to additional domains and to incorporate emerging technologies, particularly artificial-intelligence tools and virtual-reality platforms, to establish a more comprehensive information foundation for future analyses. This future analyses should also include research involved with multiple sensory modalities (auditory, tactile, olfactory and proprioceptive).

To facilitate cross-study comparison and enhance methodological transparency, the organization of Table 1 draws on the study-categorization framework proposed by Lee et al. [2], which offers a structured model for integrating research directions/concepts, expe-riential tools and environment setting in neuroarchitectural research.

The 15 empirical papers were organized into SPEC-aligned categories: somatic/physiological (e.g., autonomic and neural measures), psychological/behavioral (task performance, navigation, satisfaction), emotional/affective (valence, arousal indices), and cognitive (attention, memory, decision-making), thereby creating a cross-domain structure for cumulative interpretation (see Table 1). The thematic overlap across these domains further demonstrates that VR and AI methodologies can unify fragmented lines of inquiry into a reproducible framework for neuroarchitectural research. 

## 5. Discussion and Conclusions

The literature review reveals significant progress in the field of neuroarchitecture over the past decade. These advancements can be broadly categorized into two main trajectories: first, the deepening understanding of neural mechanisms through neuroscientific research; and second, the growing emphasis on quantitative, empirically grounded studies aimed at generating actionable insights for architectural design. This review focuses primarily on the latter, exploring how empirical findings can inform and enhance the architectural design process. One notable observation is the growing prominence of quantitative empirical methods, particularly those based on physiological measurements, as opposed to self-reported data such as questionnaires, in current research on the relationship between architecture and human experience. This shift is largely enabled by advances in immersive virtual reality (VR), which has gained validation as a credible tool for simulating spatial experience, and by the refinement of experimental protocols employing neurophysiological tools such as fMRI and EEG to record and analyze brain activity (see Table 1).To strengthen methodological transparency, reports should include: (1) the exact VR hardware and environmental parameters; (2) the preprocessing and artifact-rejection steps for physiological signals; and (3) effect sizes with 95% confidence intervals. Consistent reporting will allow quantitative comparison across studies and facilitate meta-analytic aggregation. Moreover, integrating findings within the SPEC domains offers a systematic way to interpret the combined behavioral, physiological, and affective evidence, ensuring that neuroarchitectural conclusions rest on reproducible, multi-level data rather than anecdotal observation.

Because VR can render a full-scale digital twin of a proposed lobby or streetscape, researchers can rapidly immerse users in alternative configurations, testing variations in lighting schemes, ceiling heights, or façade articulations. Findings from stress-induction studies and object-size experiments already hint at actionable rules of thumb. For example, moderate scale, visual openness, and guided sightlines mitigate sympathetic arousal.

Building on these results, future studies should report detailed VR configuration data, including headset type, frame rate, and environmental-scale calibration, to ensure that design “rules of thumb” derived from such experiments are verifiable and transferable across platforms and laboratories.

Despite recent advances in neuroarchitectural research methodologies, the body of empirical knowledge derived specifically from this field remains limited. As a conclusion derived from Table 1, this is largely due to the inherent challenges in defining, categorizing, and hierarchizing the criteria that influence different users. Consequently, while current studies follow diverse research directions, the body of knowledge we aim to develop may be grounded in clearly identifiable architectural features with demonstrable influence.

Addressing this fragmentation will require meta-analytic synthesis across SPEC domains, identifying which spatial parameters consistently modulate physiological (somatic), behavioral (psychological), emotional, and cognitive outcomes. Such cross-domain mapping would create an empirical foundation for design guidelines grounded in reproducible evidence.

While much of the current in neuroarchitecture research reviewed in the paper remains exploratory, the increasing fidelity of VR simulations and the analytical power of AI signal a shift toward translational applications in real-world design practice. AI then may convert facial cues, EEG patterns, and autonomic nervous system (ANS) markers into quantitative comfort and arousal indices. When emotion-recognition networks succeed despite headset occlusion and multimodal fusion boosts accuracy, designers may gain confidence that the data reflect genuine affect rather than measurement noise. As real-time biofeedback becomes more reliable, it may be integrated directly into computational design workflows, allowing architects to evaluate the experiential quality of space before construction begins. This suggests a future where architectural design is informed not only by structural, environmental and aesthetic logics but also by measurable neural and physiological insights.

Explicitly linking these translational pipelines to ethical standards, covering consent, data protection, and algorithmic transparency, will be essential for the responsible deployment of neuro-informed design technologies.

Building on Mostafavi’s framework, much of the literature examines how architectural settings modulate three core domains of human experience- somatic (physiological), emotional, and cognitive. We propose extending this into a four-component “SPEC” framework that explicitly incorporates a behavioral (psychological) dimension alongside these domains. In this formulation, the behavioral component captures observable actions and choices (e.g., gaze allocation, locomotion paths, approach–avoidance, task performance) that are shaped by external conditions such as social context, task demands, and environmental affordances. Conceptually, behavior often functions as a downstream manifestation, and sometimes as a mediator, of somatic, emotional, and cognitive states under specific environmental constraints.

Future validation of the SPEC model should include standardized behavioral batteries and open repositories where researchers can deposit synchronized neural, physiological, and behavioral datasets annotated by architectural variables, enabling cumulative testing and refinement of the framework.

Methodologically, contemporary AI offers a principled way to operationalize this behavioral layer and address its variability. Multimodal learning pipelines can integrate physiological signals (EEG, EDA, HRV), affective indices, and cognitive proxies (eye-tracking metrics, workload estimates) with rich behavioral traces (navigation trajectories, interaction logs) and environmental descriptors (spatial syntax, luminance/color statistics, acoustic features). Such models can capture non-linear interactions, handle individual heterogeneity via hierarchical or representation-learning approaches, and improve out-of-sample prediction of human responses to architectural space. In VR-based design reviews, this enables closed-loop, person-specific feedback- linking changes in form, layout, color, or lighting to expected behavioral outcomes- thereby addressing a key gap in current predictive validity. Care is still required around standardization, interpretability, and ethics (privacy, consent, bias), but SPEC provides a coherent scaffold for cumulative, data-driven inference about how design features shape human experience and behavior.

In line with reviewer recommendations, this paragraph explicitly ties AI modeling to reproducibility: we should in the future develop model architectures, training data summaries, and validation metrics to permit replication and meta-analysis across projects.

The findings reviewed here point toward the emergence of a neuro-informed design paradigm, one that draws systematically on brain-based data to optimize environments for well-being, performance, and social interaction. Such a paradigm could eventually give rise to new professional roles bridging neuroscience, AI, and architectural practice. It also invites the development of standardized neural evaluation protocols, akin to energy or acoustic performance ratings, which could anchor regulatory or institutional frameworks for health-promoting architecture. To realize this vision, further interdisciplinary collaboration is required to move from fragmented experimentation toward shared methodologies, validated metrics, and scalable toolkits for practice.

Embedding these standards within professional accreditation bodies and open-data consortia would accelerate adoption and ensure that neuro-informed design principles benefit diverse populations rather than remaining confined to specialized research settings.

However, a key limitation of this body of work lies in its insufficient translation into actionable tools or frameworks that can directly inform the architectural design process, despite the valuable insights it has produced. Looking ahead, one can envision the development of a new class of simulation tools capable of modeling human emotional and cognitive responses to architectural spaces, analogous to how lighting or shading simulations are currently employed by architects in the early stages of the design process.

Establishing such predictive simulation platforms should be treated as a near-term research priority, supported by open benchmarks and standardized validation datasets that align with the SPEC taxonomy.

### 5.1. Limitations

A recurring limitation across studies is the lack of diverse, representative datasets that reflect variability in age, culture, spatial familiarity, and neurodiversity. Most current experiments rely on convenience samples (e.g., university students), reducing generalizability. As AI models become central to architectural evaluation, it is critical that training data reflect global and inclusive experiences of space. Establishing open-access databases, comprising synchronized neural, physiological, and environmental data, could accelerate comparative studies and democratize innovation across institutions and geographies.

In addition, consistent metadata standards (e.g., participant demographics, spatial descriptors, acquisition hardware) should accompany shared datasets so that cross-cultural and cross-site comparisons remain interpretable and ethically sound.

### 5.2. Closing Remarks—Toward a Neuro-Informed Design Paradigm

This review highlights a growing consensus: spatial geometry, materiality, light, sound, and multisensory cues leave measurable neural and physiological signatures, confirming that architecture is an embodied experience amenable to empirical study. Progress, however, is still constrained by methodological heterogeneity, small samples, and fragmented data. A next phase of research should therefore integrate high-fidelity, openly shared datasets that pair synchronized neural and physiological recordings with detailed spatial descriptors; pursue hybrid VR-real-world experiments to ensure ecological validity. By fusing rigorous neuroscience with advanced computation and responsible design, the field can move toward developing simulation and design tools that will give designers the ability to design spaces that are not only structurally and aesthetically sound but clearly supportive of human health, cognition, and emotion across diverse populations. Implementing these measures will enable neuroarchitecture to evolve from exploratory investigation into a mature, predictive science capable of informing global design practice.

## Figures and Tables

**Table 1:** Research Directions and Tools.

Research Direction	SPEC (Somatic, Psychological, Emotional and Cognitive)	Brain Region	Experimental Tool/Procedure	Environment setting	Display (Type & Stereo)	Field of View	Frame Rate/ Latency	Reference
Different space styles	E- Emotional: affective/pleasantness responses to different architectural space styles. S- Somatic: EEG activity in PCC and occipital cortex as physiological correlates of those emotions.	Posterior cingulate cortex (PCC) and the occipital lobe	VR navigation + 128-ch EEG	VR	HMD (Samsung Gear VR for pre-test; HTC Vive for main study; stereoscopic)	~110° (Hardware specs)	90 Hz (Hardware specs)	[11]
Restorative qualities of campus environments	C- Cognitive: visual perception and allocation of attention, measured via eye-tracking, Psychological- temporal and spatial information, dynamic VR stimuli, Emotional- natural elements correlation with fascination.	-	VR; Eye-tracking; Questionnaire	VR	HTC Vive Pro2	~120° (Hardware specs)	90 Hz (Hardware specs)	[46]
Aesthetic appraisal of space geometry & material (warm-vs-cool ambience)	E- Emotional: aesthetic and affective appraisal (pleasantness) of different geometries/materials and warm vs. cool ambiences. S- Somatic: frontal-alpha asymmetry as a physiological index of affective state.	Frontal Cortex	EEG frontal-alpha asymmetry	Images	-	-	-	[72]
Influence of space geometry on users’ emotional and cognitive Reactions	E- Emotional: intensity of a physiological response generated by different room geometries. S- Somatic: Β ratio (EEG), GSR and eye-tracking measures as autonomic/neural indices. P- Psychological: questionnaire ratings, C- Cognitive- duration of presence in space.	Frontal cortex, parieto-temporal, and occipital	Β ratio (wireless EEG) Eye tracking GSR rating rendered spaces	VR	HTC Vive; wireless EEG headset (Emotive Insight)	~110°	90 Hz (Hardware specs)	[6,73]
Ceiling-height × enclosure effects on beauty ratings	E- Emotional: beauty and pleasantness ratings of ceiling height and enclosure. P- Psychological: approach–avoidance tendencies linked to those evaluations. C- Cognitive and Somatic: evaluative and decisional processes in IPS during aesthetic judgments.	Anterior midcingulate cortex (aMCC)	fMRI while rating rendered rooms	Images	-	-	-	[74]
Architectural affordances (doorway width)	P- Psychological: action tendencies and approach–avoidance behaviors in relation to doorway width. C- Cognitive: prediction of action possibilities and spatial decision-making (affordance-based cognition). S- Somatic: visual and premotor EEG dynamics reflecting sensorimotor preparation.	Visual cortex and the motor cortex	64-ch mobile EEG	VR with walking	HMD (Windows Mixed Reality headset; stereo)	~100°	90 Hz	[43]
Lighting conditions	E- Emotional: mood and affective state changes under different simulated lighting conditions. S- Somatic: EEG changes as physiological markers of lighting-induced affect.	-	40-channel Quik-cap	Real world		-	-	[75]
Architectural styles	C- Cognitive: categorical recognition and representation of architectural styles in high-level visual areas (FFA, PPA, LOC). S- Somatic: fMRI activity patterns as physiological signatures of style processing.	Fusiform face area (FFA), PPA, LOC	fMRI	Static images of buildings	-	-	-	[76]
Perceived spaciousness	P- Psychological: subjective sense of spaciousness and comfort in different layouts. C- Cognitive: visual perception and allocation of attention, measured via eye-tracking patterns in VR images.	-	VR; Eye-tracking	360-degree panoramic views	Meta Quest Pro wireless VR headset; Xreal Air 2 augmented reality (AR) glasses	~100°	90 Hz	[77]
Rectangular vs. curved room	E- Emotional: affective state changes in rectangular vs. curved rooms. S- Somatic: heart-rate variability (HRV) as autonomic indicator of stress/relaxation. C- Cognitive: creative performance differences (idea generation, task output) across room geometries.	-	HRV; Self-report questionnaire	VR	Vive VR headset	-	-	[78]
Cityscape	C- Cognitive: visual processing and attention distribution across façades and streetscapes in VR. P- Psychological: evaluation, preference and perceived quality/protection of different cityscape configurations.	-	Eye-tracking	VR	HTC Vive; 7Invensun aGlass eye tracker	~110° (Hardware specs)	90 Hz (Hardware specs)	[79]
Biophilic design	E- Emotional: emotional and restorative effects of biophilic vs. non-biophilic VR rooms (e.g., hospital settings). S- Somatic: EEG and autonomic responses to biophilic elements. C- Cognitive: changes in attentional engagement and processing of biophilic environments.	Frontal region	EEG; Self-report questionnaire	VR	HTC Vive Pro; wireless EEG headset (Emotive)	~110° (Hardware specs)	90 Hz	[21]
Forest density	P- Psychological: perceived safety, comfort and preference at different forest densities. E- Emotional: restorative/affective responses to varying levels of visual openness. C- Cognitive: perception of permeability, legibility and visual access as reflected in gaze behavior.	-	Eye-tracker	VR	HTC Vive Pro; Ergo VR	≥120° tracking range	90 Hz (Hardware specs)	[47]
Natural indoor environments	E- Emotional: Self-reported relaxation and emotional valence. S- Somatic: Neural activity measured via EEG frequency-band ratios. C- Cognitive: Executive function and attention assessed through Stroop, Go/No-Go, and Error Detection tasks.	Frontal and occipital regions	14-channel EEG headset	VR	Meta Quest 2	~100°	90 Hz	[36]
Street space design (interface types and green ratings)	S- Somatic: EEG spectral bands and their ratios reflecting brain activation states. E- Emotional: healing measures derived from EEG indicators correlated with subjective comfort reports. C- Cognitive: load and engagement inferred from EEG ratios indicating attention and information processing.	Occipital, frontal, temporal, parietal, central, and motor regions	64-channel EEG and remote eye-tracking	Images	*aSee Pro* remote eye-tracking system	-	Eye-tracking- 140 Hz	[24]

## Data Availability

No new data were created or analyzed in this study.
